# The Role of a Loop in the Non-catalytic Domain B on the Hydrolysis/Transglycosylation Specificity of the 4-α-Glucanotransferase from *Thermotoga maritima*

**DOI:** 10.1007/s10930-023-10136-2

**Published:** 2023-07-18

**Authors:** Alexey Llopiz, Marco A. Ramírez-Martínez, Leticia Olvera, Wendy Xolalpa-Villanueva, Nina Pastor, Gloria Saab-Rincon

**Affiliations:** 1 Departamento de Ingeniería Celular y Biocatálisis, Instituto de Biotecnología, Universidad Nacional Autónoma de México, 62209 Cuernavaca, Morelos Mexico; 2 Centro de Investigación en Dinámica Celular, IICBA, Universidad Autónoma del Estado de Morelos, 62209 Cuernavaca, Morelos Mexico

**Keywords:** Hydrolysis, Transglycosylation, Glycosidases, Glucanotransferase, Reaction-specificity

## Abstract

The mechanism by which glycoside hydrolases control the reaction specificity through hydrolysis or transglycosylation is a key element embedded in their chemical structures. The determinants of reaction specificity seem to be complex. We looked for structural differences in domain B between the 4-α-glucanotransferase from *Thermotoga maritima* (*Tm*GTase) and the α-amylase from *Thermotoga petrophila (Tp*Amylase*)* and found a longer loop in the former that extends towards the active site carrying a W residue at its tip. Based on these differences we constructed the variants W131G and the partial deletion of the loop at residues 120-124/128-131, which showed a 11.6 and 11.4-fold increased hydrolysis/transglycosylation (H/T) ratio relative to WT protein, respectively. These variants had a reduction in the maximum velocity of the transglycosylation reaction, while their affinity for maltose as the acceptor was not substantially affected. Molecular dynamics simulations allow us to rationalize the increase in H/T ratio in terms of the flexibility near the active site and the conformations of the catalytic acid residues and their associated p*Ka*s.

**Supplementary Information:**

The online version contains supplementary material available at 10.1007/s10930-023-10136-2.

## Introduction

**Fig. 1:**
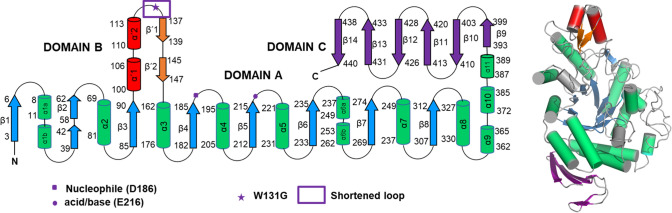
Topological map of secondary structure elements (left panel) and 3D-structure (right panel) of *Tm*GTase (PDB ID 1LWJ). α-Helices are represented by rods and β-strands are indicated as arrows. Domain A: green and blue; Domain B: red and orange; Domain C: purple (Color figure online)

Glycoside hydrolases (GHs) catalyze the degradation of polysaccharides such as starch and glycogen, energy reservoirs widely used by living organisms, and other glycosyl substrates. These enzymes are broadly distributed in bacteria, fungi, yeasts, plants, and animals and have important biological, industrial, and medical applications [1]. A particular group of GHs is family 13 also known as alpha-amylases (EC 3.2.1.1). This family comprises a large group of starch hydrolases with at least 20 different specificities [2, 3], some of which catalyze hydrolysis and transfer reactions of α-D-glycosidic linkages. Glucanotransferases (EC 2.4.1.25), on the other hand, transfer the remainder of the glycoside to another glycoside rather than to water after cleavage of the glycosidic bond. Many members of this family of enzymes catalyze both reactions with a bias dependent on the specific enzyme [4]. For example, the α-amylases of *B. licheniformis* and *B. stearothermophilus* are exclusively hydrolytic [5–7], while others, such as the 4-α-glucanotransferase of *Thermotoga maritima* (*Tm*GTase) are predominantly transglycosidic [8]. However, there is also a group within the GH13 family in which both reactions are present and compete with one another. These include α-amylase from *T. maritima* [9, 10], cyclodextrin glucanotransferase NO2 from *B. stearothermophilus* [11, 12] and Amyrel (amylase from *Drosophila melanogaster*) [13]. GH13 family glycosidases share a similar core 3D- structure, comprising three domains: Domain A formed by a (β/α)_8_ barrel catalytic domain, which is interrupted by the smaller and more variable domain B between the third β-strand and the third α-helix, and domain C at the end of domain A, with a Greek key structure. Besides sharing the catalytic architecture, and reaction mechanism, some of them, mainly those with transglycosidase activity, have a variable number of extra domains either at the N- or at the C- terminus, some acknowledged as carbohydrate binding domains and some with unknown function [14, 15] (Fig. 1). Domain A is the major contributor to the activity of the protein, but domain B acts as a clamp for binding the carbohydrate chain [16, 17]. The binding site of these proteins consists of a number of subsites, each of which binds one glucose unit. The subsites are numbered according to the glycosidic bond cleaved so that positive subsites are assigned to those belonging to the released saccharide, which also constitute the acceptor binding site in transglycosidic enzymes. Negative subsites, on the other hand, are those belonging to the part of the molecule that remains covalently bound to the enzyme and is transferred in the second reaction step [18].

**Fig. 2:**
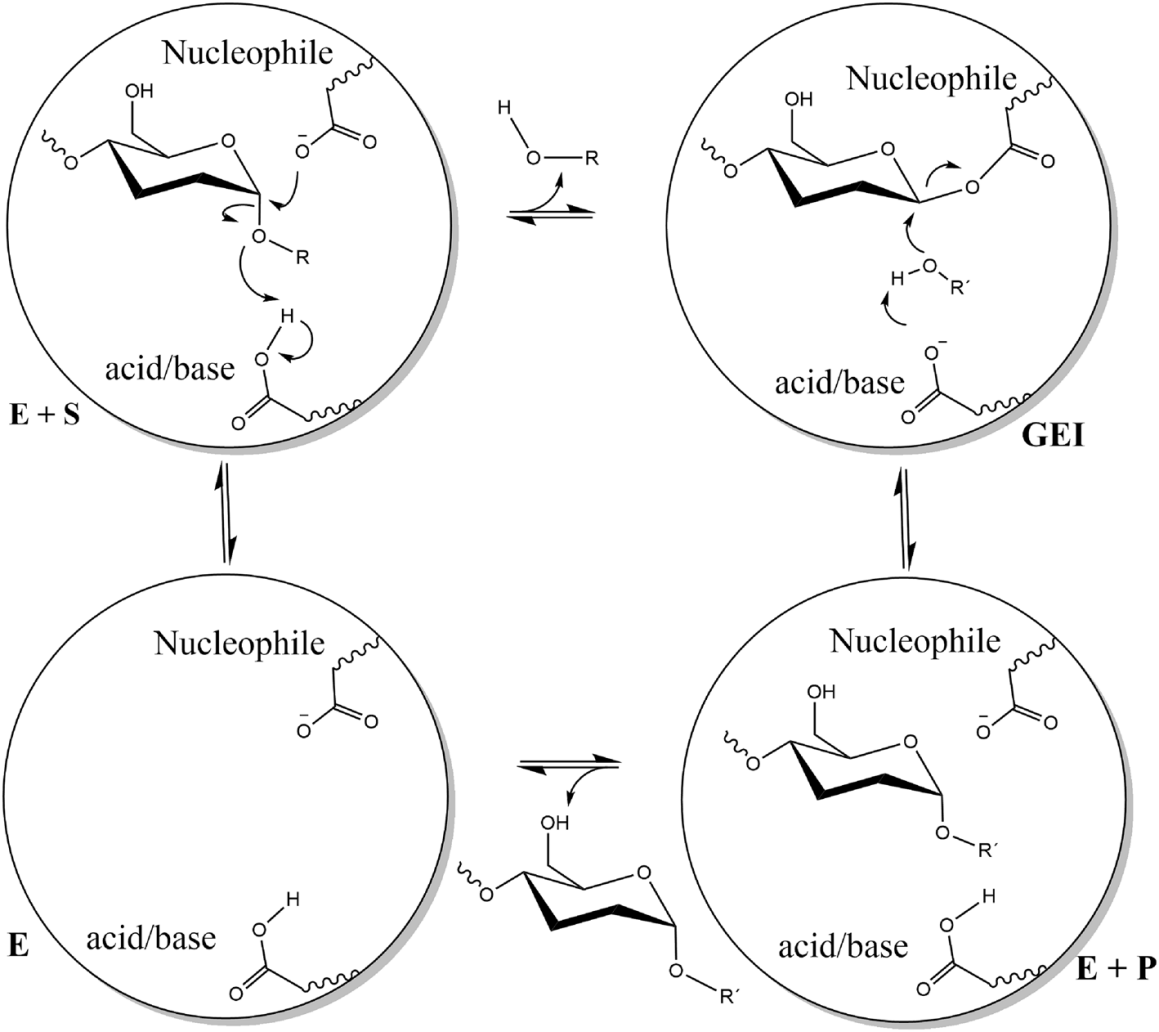
General mechanism of glycosyl hydrolase-catalyzed hydrolysis (R′ = H) or transference (R′ = Glycosyl, alkyl)) reactions produced with retaining stereochemical configuration of anomeric carbon

The reaction catalyzed by many GHs is carried out by a classical Koshland double displacement mechanism with configuration retention of the anomeric carbon at the cleavage site [19]. The initiating event in this mechanism is a hydrogen transfer from an acid residue to the leaving group. Simultaneously, the nucleophile attacks the anomeric carbon, forming a covalent bond between the enzyme and the remaining glycoside moiety, forming a glycosyl-enzyme intermediate (GEI) [20, 21]. In the second stage, an incoming nucleophile is activated by the same residue that acts as the base by removing the proton. The activated nucleophile then attacks the GEI, releasing the end product and leaving the enzyme ready to start a new cycle (Fig. 2). The nucleophilic acceptor could be a water molecule (hydrolysis), or a molecule different from water (transglycosylation), such as another sugar, an alcohol [22, 23], a phenol [24], a carboxylic acid [4] or even an amine [25]. Usually, transglycosylation shows lower rates than hydrolysis in members of GH13. Understanding the mechanisms and structural elements by which many glycoside hydrolases control their preferences for hydrolysis or transglycosylation is important for manipulating or designing enzymes with potential applications. However, progress toward this goal has been hampered by the inherent complexity of the process [26]. Some of the phenomena associated with the specificity of the reaction are the presence of a flexible water channel from the protein surface to the active site [27], changes in the p*Ka* of catalytic residues [6, 28], the presence of hydrophobic residues at the binding subsites [29], the absence/presence of loops facing towards the active site associated to product release [13, 30–32], and the protein dynamics around the active site [28, 29, 33–35].

There has been much interest in modifying these enzymes to change their reaction preference, most commonly to increase transglycosylation, for glycosynthesis purposes. The search for mutation sites is generally based on the sequence alignment of the regions in the vicinity of the active site. Due to its high sequence conservation, most of these sites are located in the catalytic domain (domain A). Changes in the domain B are less easy to predict, as this is the most variable domain in this family of proteins; however, evidence of its involvement in determining reaction specificity can be found in the amylosucrases from *Deinococcus geothermalis* and *Neisserria polysaccharea* [36], the *Listeria monocytogenes* cycloalternan forming enzyme (*Lm*CAFE) [29] and *Thermoanerobacterium thermosulfurigenes* cyclodextrin glycosyltransferase (CGTase) [37]. Most of the mutations involve aromatic/hydrophobic residues, at both the acceptor (positive) and donor (negative) [38, 39] subsites, which discreetly increase the affinity for carbohydrates while at the same time disfavor the positioning of water molecules [4, 11, 27, 40–42].

**Fig. 3:**
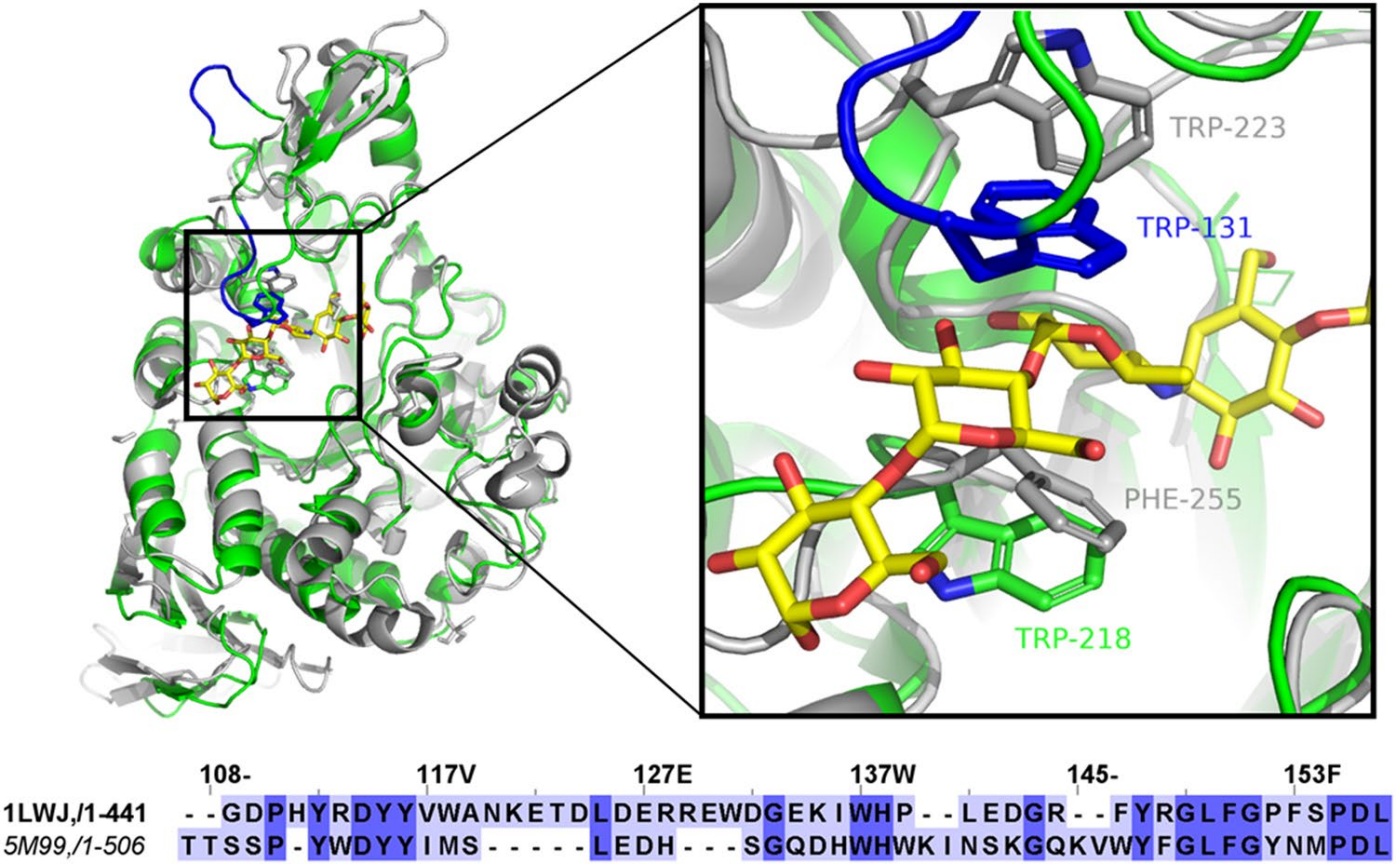
Sites targeted to change the reaction specificity in the *Tm*GTase. Differences in the targeted loop from domain B of *Tm*GTase (PDB ID 1LWJ, green), and *Tp*Amylase (PDB ID 5M99, gray). The differential part of the protruding loop (shown in blue) with Trp131 in the tip is present only in the transferase enzyme. The competitive inhibitor acarbose –a delimiter of the active site– is presented as yellow sticks. The sequence alignment between *Tm*GTase and *Tp*Amylase for the loop region is presented in the lower panel (Color figure online)

In the present work we used the *Tm*GTase as a model to study the role of structural elements in domain B on reaction specificity. The 53 kDa protein is an unclassified member of the GH13 family, composed of 441 amino acid residues, that has been crystallized in the presence and absence of the competitive inhibitor acarbose [43]. This protein is a classical transglycosidic enzyme with negligible hydrolytic activity, which requires at least maltose as an acceptor for the transfer of glucose units from starch [44]. Structural comparison of *Tm*GTase with the α-Amylase from *T. petrophila* (*Tp*Amylase) belonging to subfamily GH13_36, reveals a long loop with a W residue in the tip extending over subsite + 2 in *Tm*GTase (Fig. 3), which is considerably shorter in the hydrolase. To investigate the role of this extended loop, we constructed two variants of this enzyme, the first with a nine amino acid residues deletion to shorten the loop and the second with W131 replaced by G to remove a functional group. Both variants showed altered hydrolytic and transglycosidic activities when we carried out kinetic assays. We performed molecular dynamics simulations to propose a mechanism mediating the change in reaction specificity.

## Materials and Methods

### Materials

All used reagents were for analysis grade. Yeast extract, tryptone, and IPTG (isopropyl-β-D-thiogalactopyranoside) were obtained from Thermo Fisher Scientific (USA). Sodium chloride, sodium phosphate, and magnesium chloride were acquired from J.T.Baker (USA). Lysozyme, ampicillin, imidazole, calcium chloride, soluble starch, DNS (Dinitro Salicylic acid), iodine species, and Tris-HCl were from Sigma (USA). Maltose was purchased in Research Organics (USA).

### Mutation Site Selection

The 3D-structures of the 4-α-glucanotransferase from *T. maritima* (PDB ID 1LWJ) [43] and the amylase from *T. petrophila* (PDB ID 5M99) [45] were downloaded from PDB (https://www.rcsb.org/) [46] and compared by using the MatchMaker algorithm implemented in UCSF Chimera [47]. The superposed structures were visualized and analyzed in Chimera, and the derived alignment was seen with Jalview [48]. The sequence-specific regions (SSR) were computed with the algorithm Zebra3D [49]. The run was carried out with the structure 1LWJ from PDB as a query on mode 4 (Mustguseal + Zebra3D) of Mustguseal server [50, 51].

### Construction of *TmGTase* Mutants

The *mgtA* gene from *T. maritima* (GenBank accession number AAD35451.1), previously cloned in the plasmid pET22b(+) [52], was used as a template to build the two mutants of *Tm*GTase. The mutant genes were constructed by PCR using the corresponding pair of oligonucleotides for each mutation shown in Table 1. The first half of the *Tm*GTase gene was constructed using a *mgtA* containing pET22b(+) plasmid as template and the oligonucleotide T7_Fw as 5′-primer with a *Nde*I restriction site and the respective mutagenic noncoding oligonucleotide as 3′-primer. The second half was constructed using the same plasmid as template and their corresponding coding mutagenic oligonucleotide as 5′-primer and the oligonucleotide *Xho*I_term_Rv with a *Xho*I restriction site as 3′-primer (Table 1). The amplification products were purified from a 1% agarose gel with the *High Pure PCR Extraction Purification kit* (Roche Diagnostics GmbH, Germany). Mutated genes were finally constructed by overlapping extension PCR using the corresponding two PCR products from the previous reactions as templates and the oligonucleotides T7_Fw as 5′-primer and *Xho*I_Term_Rv as 3′-primer. Afterward, amplified products were purified from a 1% agarose gel and then digested with *Nde*I and *Xho*I, and the purified reaction products were ligated into the pET22b(+) plasmid previously digested with the same restriction enzymes and purified. This plasmid adds a His-tag at the C-terminus of the protein. Plasmid and PCR products were ligated with T4 DNA ligase according to manufacturer recommendations (Thermo Scientific, USA) and finally, electroporated in competent *E. coli* MC1061 cells. The culture obtained after one hour of incubation was plated on a solid LB medium containing 200 µg/mL ampicillin and incubated at 37 °C for 12 h. All plasmid isolations were performed using the *High Pure Plasmid Isolation kit* (Roche Diagnostics GmbH, Germany). The *mgtA* gene was sequenced completely to ensure that mutations other than those designed did not occur. DNA quantification was accomplished by measuring the absorbance at 260 nm in a *NanoDrop* 2000 (Thermo Fisher Scientific, USA). The design of oligonucleotides and the analysis in silico were performed with SnapGene (GSL Biotech LLC, USA).

**Table 1:** Primers used for site-directed mutagenesis of *Tm*GTase

Mutant	Fragment		Primer	Sequence (5′-3′ direction)			
W131G	F1		T7_Fw	CTT TAA GAA GGA GAT ATA CAT ATG ATA GGC TAT CAG			
			W131G_Rv	CTT TTC TCC ATC CCC CTC TCT TCT TTC GTC C			
	F2		W131G_Fw	G GAC GAA AGA AGA GAG GGG GAT GGA GAA AAG			
			XhoI_Term_Rv	GCTAGTTATTGCTCAGCGG			
Truncated loop	F1		T7_Fw	CTTTAAGAAGGAGATATACATATGATAGGCTATCAG			
			SL_Rv	GATCTTTTCTCCATCTTCGTCCAGTGCCCATACGTAGTAATCTCTGTAG			
	F2		SL_Fw	CTACAGAGATTACTACGTATGGGCACTGGACGAAGATGGAGAAAAGATC			
			XhoI_Term_Rv	GCTAGTTATTGCTCAGCGG			

### Protein Expression and Purification

BL21 cells transformed with plasmids carrying the respective mutations were cultured at 37 °C in LB medium supplemented with 200 µg/mL ampicillin. Protein expression was induced when the absorbance at 600 nm of cultures was between 0.5 and 0.6 units by the addition of IPTG (isopropyl-β-D-thiogalactopyranoside) to a 0.25 mM final concentration, and incubation continued at 20 °C for 16 h. Biomass was obtained by centrifugation at 1507 g at 4 °C for 30 min in a 5804R refrigerated centrifuge (Eppendorf, Germany). For initial analysis, the cellular disruption was carried out in 100 mM sodium phosphate pH 8.0 containing 0.75 mg/mL lysozyme, 21 mM MgCl_2,_ and 20 µg/mL DNAse, incubating for 1 h at 37 °C with a shaker at 200 rpm. Later, insoluble material was separated through centrifugation at 1507 g at 4 °C for 30 min in a 5804R centrifuge (Eppendorf, Germany).

For protein purification, *E. coli* cells transformed with the plasmids containing the genes of interest were grown in 1 L of LB medium, and protein expression was induced with IPTG as described above. The cell pellets obtained after centrifugation were resuspended in 300 mM NaCl, 50 mM Na_2_HPO_4_, pH 7.7 (*Buffer* A), and lysed by sonication on an ice bath (Branson Sonifier 450, Emerson Inc., USA). Afterward, the soluble extracts were heated at 70 °C for one hour, and the insoluble material was eliminated by centrifugation at 1507 g at 4 °C for 30 min in a 5804R centrifuge (Eppendorf, Germany). Protein purification was accomplished in Ni Sepharose High Performance (GE Healthcare, USA) affinity matrix, previously equilibrated in *Buffer A*. The column was washed first with 20 CV of *Buffer A* and then with an equal volume of *Buffer A* containing 15 mM imidazole. Finally, the protein was eluted with 10 mL of 300 mM imidazole in 300 mM NaCl, 50 mM Na_2_HPO_4_, pH 7.7 (*Elution Buffer*). Fractions with higher absorbance at 280 nm and high enzymatic (transglycosidic) activity were pooled, concentrated, and dialyzed overnight against 50 mM Tris buffer, NaCl 150 mM, pH 7.5. The purity of proteins was assured by 15% SDS-PAGE following the procedure described by Laemmli [53]. The gels were stained with Coomassie Brilliant Blue R-250 to visualize the proteins. Densitometric analysis to estimate the % of the full-length protein was carried out with Image Lab software v 6.1 (BioRad, USA).

### Transglycosylation Assay

The transglycosylation reaction was based on the method reported [8] with modifications to ensure saturating conditions. Briefly, 90 µL of the mixture containing 0.9% starch, and varying maltose concentrations from 1.2 to 20.5 mM in 50 mM Tris/HCl, 150 mM NaCl, pH 7.0 buffer, and 3 µg of purified enzymes were incubated at 70 °C for five to fifteen minutes to ensure measurement of initial velocity. Reactions (20 µL) were stopped with 0.4 M NaOH (10 µL) and subsequently neutralized with an equal volume of 0.4 M HCl. Later, each solution was diluted 25-fold with ultrapure water, and to quantify the remaining starch, each reaction was mixed with 100 µl 0.02% iodine/potassium iodide solution (Lugol’s solution, diluted 1:50 with 50 mM Tris/HCl, 150 mM NaCl, pH 7.0 buffer), and the complex between the remaining starch and triiodide was monitored by the absorbance at 620 nm on a microplate reader Saphire 2 (TECAN, USA). The absorbances were subtracted from the values of the corresponding zero-time samples. One unit of GTase activity was arbitrarily defined as the amount of enzyme which causes a change in one absorbance unit per unit time under the above conditions. In the case of W131G, the data were corrected by the percentage of full-length protein in the sample.

### Hydrolysis Assay

Hydrolysis reactions were performed with 1% starch in 50 mM Tris/HCl, 150 mM NaCl, pH 7.0, starting the reaction by the addition of 3 µg of purified enzymes and incubated for 12 h at 70 °C. The hydrolysis products were measured as reducing sugars using the dinitrosalicylate (DNS) method [54]. In all cases, the absorbance at 540 nm was measured using a microplate reader Saphire 2 (TECAN, USA). A standard glucose curve was prepared under the same conditions to compare the amount of reducing sugars, and the values are expressed as dextrose equivalents per volume unit. The values were corrected by subtracting the reducing sugars obtained from a sample incubated under the same conditions but without enzyme. A unit of enzyme activity is defined as the glucose equivalents (mol) released per min and is reported in terms of mg of protein. In the case of W131G, the data were corrected by the percentage of full-length protein in the sample.

### Determination of p*Ka* for the Catalytic Residues

The influence of varying pH values on *Tm*GTase activity was determined at 70 °C using a mixture of citrate, phosphate, and glycine buffers (100 mM each one) to cover the pH range from 3.0 to 11.0. The assays were performed as described above using 0.5 U/mL of purified enzymes and measuring the difference in absorbance at 620 nm after 15 min of reaction as was described [43]. The data were fit to the following equation to evaluate the p*Ka* of the active site residues, using Kaleida Graph V 3.5:

1 $$\% \;Activity\, = \,\frac{1}{{1\, + \,10^{{pK_{a1} - pH}} \, + \,10^{{pH - pK_{a2} }} }}$$

However, the wild-type (WT) enzyme showed a polyprotic behavior, so that the following model was considered to fit the data:

$${\text{EH}}_{4} ~\quad \mathop \Leftrightarrow \limits_{{Ka1}} \quad {\text{EH}}_{{\text{3}}}^{ - } {\mkern 1mu} + {\mkern 1mu} {\text{H}}^{ + } \quad \mathop \Leftrightarrow \limits_{{Ka2}} \quad {\text{EH}}_{{\text{2}}}^{ 2- } {\mkern 1mu} + {\mkern 1mu} {\text{H}}^{ + } \quad \mathop \Leftrightarrow \limits_{{Ka3}} \quad {\text{EH}}^{{{\text{3}} - }} {\mkern 1mu} + {\mkern 1mu} {\text{H}}^{{ + ~}} \quad \mathop \Leftrightarrow \limits_{{Ka4}} \quad {\text{E}}^{{{\text{4}} - }} {\mkern 1mu} + {\mkern 1mu} {\text{H}}^{ + }$$

where species EH_3_^−^, EH_2_^2−^, and EH^3−^, are considered to have x, y, and z relative activity, respectively. According with this model the WT data was also fit using the following equation:

2 $$\% \;Activity\, = \,\frac{{x\frac{{\left[ {H^{ + } } \right]}}{{K_{{a2}} }}\, + \,y\, + \,z\frac{{K_{{a3}} }}{{\left[ {H^{ + } } \right]}}}}{{\frac{{\left[ {H^{ + } } \right]^{2} }}{{K_{{a1}} K_{{a2}} }}\, + \,\frac{{\left[ {H^{ + } } \right]}}{{K_{{a2}} }}\, + \,1\, + \,\frac{{K_{{a3}} }}{{\left[ {H^{ + } } \right]}}\, + \,\frac{{K_{{a3}} K_{{a4}} }}{{\left[ {H^{ + } } \right]^{2} }}}}$$

For the W131G variant the following model was used to explain its complex behavior:

$${\text{EH}}_{{\text{3}}} \quad \mathop \Leftrightarrow \limits_{{Ka1}} \quad {\text{EH}}_{2}^{ - } {\mkern 1mu} + {\mkern 1mu} {\text{H}}^{ + } \quad \mathop \Leftrightarrow \limits_{{Ka2}} \quad {\text{EH}}^{ 2- } {\mkern 1mu} + {\mkern 1mu} {\text{H}}^{ + } \mathop \Leftrightarrow \limits_{{Ka3}} \quad {\text{E}}^{3-} {\mkern 1mu} + {\mkern 1mu} {\text{H}}^{ + }$$

where species EH_2_^−^, EH^2−^, are considered to have x, and y relative activity, respectively. According with this model the W131G pH profile data was also fit using the following equation:

3 $$\% \;Activity\, = \,\frac{{x\, + \,y\frac{{\left[ {H^{ + } } \right]}}{{K_{{a2}} }}}}{{\frac{{\left[ {H^{ + } } \right]^{2} }}{{K_{{a1}} K_{{a2}} }}\, + \,\frac{{\left[ {H^{ + } } \right]}}{{K_{{a2}} }}\, + \,1\, + \,\frac{{K_{{a3}} }}{{\left[ {H^{ + } } \right]}}}}$$

### CD Spectra

The CD spectra were registered in a Jasco J-710 spectropolarimeter (Jasco, USA) equipped with a Peltier temperature control using a 1.00 mm path length cell. Three CD scans were recorded and averaged from 190 to 260 nm for each sample containing between 0.02 and 0.3 mg/mL of *Tm*GTase. The buffer used was a 50 mM Tris, 150 mM NaCl, pH 7.0. The CD spectra were analyzed with the Dichroweb using the algorithm CDSTTR with set 4 [55].

### Molecular Dynamics Simulations

The atomic coordinates for *Tm*GTase (PDB ID: 1LWJ) [43] were obtained from the Protein Data Bank (https://www.rcsb.org/). The structure files were visualized and analyzed with UCSF Chimera [47], VMD [56], or Pymol (Schrödinger, Inc., USA). The truncated loop mutant structure was modeled with Swiss-Model [57] using the crystallographic structure of *Tm*GTase (PDB ID 1LWJ) as a template [43]. The quality of the resulting model was assessed with the “Structure Assessment” tools available at the Swiss-Model server (https://swissmodel.expasy.org/assess). The single mutant (W131G) and the protonation of D186 were carried out in the CHARMM-GUI server [58–60]. The charmm36m force field [60] was used for energy minimizations and the molecular dynamics (MD) simulations. All proteins were minimized using CHARMM47 [61] with steepest descent for 50 steps for hydrogen atoms only, followed by 100 steps of conjugate gradient minimization for the whole protein. Subsequently, hydration was performed using explicit water molecules with the TIP3P model in a cubic box with an edge distance of 15 Å from the surface of the protein. The systems were neutralized with 150 mM NaCl. Correction for WYF interactions and hydrogen mass partitioning were included [61]. Before MD production, an unrestrained minimization of 5000 steps was performed for the entire system using the steepest descent algorithm, followed by solvent equilibration in the NVT ensemble for one nanosecond, followed by a nanosecond-long unrestrained NPT MD simulation for the complete system. The temperature was set to 343 K and pressure at 1 atm. The van der Waals interactions were calculated using a 12 Å cutoff with a force-based switching function. The electrostatic interactions were computed by the particle-mesh Ewald method [61] with a mesh size of 12 Å. The LINCS algorithm [62] was used to constrain bond lengths involving hydrogen atoms, and the simulation step was set to 4 fs. Constant temperature was controlled by the Nose-Hoover thermostat and pressure with the Parrinello-Rahaman barostat with a compressibility of 4.5 × 10^−5^. The simulations were run with GROMACS (GROningen MAchine for Chemical Simulation) version 2020.4 [63] as three independent replicas for 400 ns each.

The p*Ka* values for the active site and surrounding residues were calculated with PROPKA 3.5.0 [64] for the last frame of every non-overlapping 100 ps simulation interval using default PARSE charges. The Root Mean Square Fluctuations (RMSF) calculated for alpha-carbons for non-overlapping 100 ps intervals, the Root Mean Square Deviation (RMSD) of alpha carbon atoms with respect to the starting structure of each MD run, and structural clustering with a cutoff of 2 Å over alpha carbon atoms were calculated with GROMACS inbuilt tools. Hydrogen bonds (heavy atom-heavy atom cutoff at 3.4 Å and no angle restriction), and carbon atom-carbon atom contacts between a protruding loop of domain B and the rest of the protein were computed with CHARMM47 with a cutoff of 6 Å.

### Statistical Analysis

Student’s *t*-test was performed using the GraphPad Prism software package v8.0.2 (GraphPad Software Inc., USA). A parametric test was used, and a Gaussian distribution for the data was assumed. The velocity vs. [maltose] was fit to the Michaelis Menten equation. The estimation of experimental p*Ka* was achieved by successive iteration based on Eqs. 1, 2, and 3.

## Results

### Identification of Mutation Sites Based on Structural Analysis

A structural alignment comparison between *Tm*GTase, a transglycosidic enzyme, and the hydrolytic α-amylase from *T. petrophila (Tp*Amylase*)* was performed. Despite low sequence identity between these proteins (33.56%), comparison at the 3D-structural level showed a RMSD value of 0.997 Å over superimposable residues. We found a difference at the entrance of the active site comprising a loop from residues 120 to 142 (numbering as presented in *Tm*GTase), which is substantially shorter in *Tp*Amylase, as shown in Fig. 3 (right panel). This loop located between helix α’2 and strand β’1 (Fig. 2) has a W residue in its tip pointing towards the active site and forming a clamp together with W218 between subsites + 1 and + 2. In the case of the *Tp*Amylase, W223 in a nearby position is provided by the loop connecting strand β4 and helix-α4 in the TIM barrel. However, this W residue shows a different orientation, pointing toward the glycosidic bond to be cleaved. The sequence alignment of the *Tm*GTase *and Tp*Amylase in this region shows insertions in the 4-α-glucanotransferase around the residues 125–128 (Fig. 3, lower panel).

Zebra software was used to identify the relevance of certain parts of the structure of *Tm*GTase in its characteristic activity. This bioinformatic tool identifies subfamily-specific regions (SSRs) such as 3D-determinants of catalytic activity that are equivalent within families/subfamilies [49]. Coincidentally, a region comprising the residues 118–154 was identified as relevant with a *Z*-score of 4.3 and a *p-value* of 9.3⋅10^−6^. It thus can be related to functional diversity and function-related dynamical events. Based on these analyses, a mutant intended to shorten the loop was constructed by deleting residues 120–124 and 128–131 from the loop in domain B (referred to as truncated loop from now on) to evaluate the effect of this region on the reaction specificity. Residues 125–127 were left due to their conservation according to the alignment shown in Fig. 3. Additionally, to investigate the role of W131 at the tip of the loop, the mutant W131G was constructed.

### Hydrolysis/Transfer Ratio Changes in *Tm*GTase

The effect of mutations on the loop comprising residues 120–131 of *Tm*GTase was investigated by measuring their transglycosidic and hydrolytic activities. *Tm*GTase has been reported as an α-transglycosidase that acts over starch in the presence of at least two units of glucose (maltose) as an acceptor molecule.

The reaction catalyzed by *Tm*GTase follows a ping-pong mechanism in which starch is the first substrate. After forming a covalent complex with the enzyme, the active site is restituted by the transference of the glycosyl moiety to an acceptor with at least two units of glucose, like maltose. The kinetic analysis carried out by varying the maltose concentration while keeping the starch concentration constant permits the estimation of the apparent maximum velocity and the affinity for the acceptor molecule. The Michaelis-Menten curves of *Tm*GTase variants showed a ten-fold reduction in the maximal velocities of the reactions (Fig. S1), while the Km for maltose (G2), unexpectedly was reduced by approximately 50%, as shown in Table 2. The W131G protein concentration was corrected for the percentage of full-length protein remaining before the analysis, estimated by densitometry of SDS-PAGE (Fig. S2).

**Table 2:** Kinetic parameters of *TmGTase* variants. Values are represented as the average ± standard deviation of at least three experimental replicates

Protein variant	Hydrolysis (H) (Eq Glucose/L*min*mg enzyme)	Transglycosylation (T) (U/(min*mg enzyme))^a^	Km^T^ (mM)	Vmax^T^/Km^T^(U/(min*mM maltose*mg enzyme)	H/T	Increment H/T (relative to WT)
WT protein	(2.52 ± 0.58)‧10^–4^	5723 ± 423	11.9 ± 1.8	400 ± 90	4.4‧10^–8^	
Truncated loop	(3.09 ± 0.58) ‧10^–4^	615 ± 34	4.7 ± 1.0	130 ± 35	5.0‧10^–7^	11.4
W131G	(3.26 ± 1. 7) ‧10^–4^	638 ± 65	6.3 ± 1.5	101 ± 35	5.1‧10^–7^	11.6

^a^A unit of activity is defined as the amount of enzyme required to decrease 1 A.U. at 620 nM per unit time

The hydrolysis reaction, measured as reducing sugars, showed only a discrete increment of around 20% for both variants. Considering the high dispersion obtained for the W131G Vmax values, the 20% increment is not significantly different from the WT protein. These data together contribute to an 11-fold higher H/T ratio for both variants relative to the WT enzyme (Table 2). The 90% loss of transglycosidic activity upon a single amino-acid replacement indicates the relevance of the W131 residue for catalysis or stability, contributing by about 1.5 kcal/mol to the stabilization of the transition state. Interestingly, during repetition experiments, we noticed that this variant lost activity rapidly, so we investigated its stability.

### Structural Changes Associated with Mutations in *Tm*GTase

**Fig. 4:**
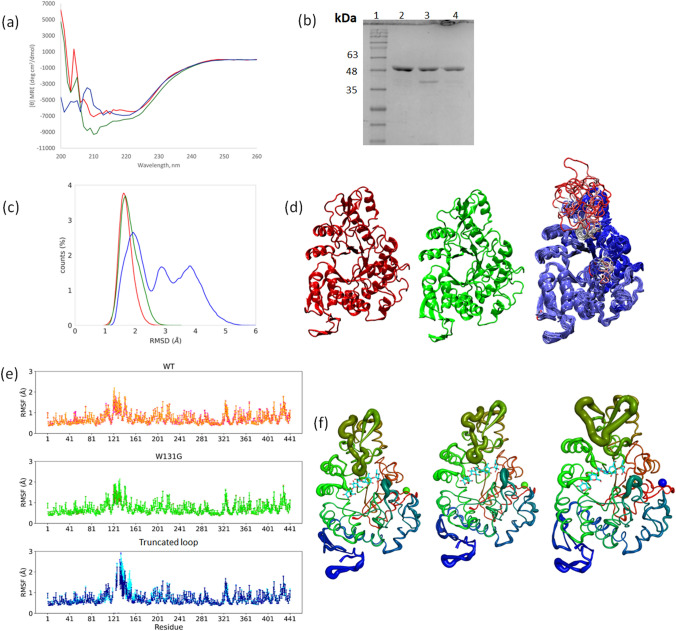
Structural characterization of *Tm*GTase variants. **a** CD spectra for *Tm*GTase WT (red), W131G (green), and truncated loop (blue) **b** SDS-PAGE of purified proteins lane 1 MW marker, lane 2 WT, lane 3 W131G and lane 4 truncated loop *Tm*GTase variants **c** RMSD distribution of WT (red), W131G (green), and truncated (blue) *Tm*GTase, **d** Representative cluster centers containing at least the 98% of the population for WT (red), W131G (green) and truncated (blue; more labile regions are shown in red). **e** Fluctuation of *Tm*GTase in 100 ps intervals. RMSF for WT (upper panel), W131G (middle panel), and truncated loop variant (lower panel). **f** Structural representation with cartoon putty from Pymol based on RMSF values of WT (left), W131G (middle), and truncated loop variant (right). The acarbose (cyan stick) indicates the active site, placed by grafting from the original 1LWJ PDB structure. All structures are colored from N-terminus (red) to middle (green) to C-terminus (blue) (Color figure online)

To determine if the mutations perturbed the protein structure or its dynamics, Molecular Dynamics Simulations (MD) and Circular Dichroism (CD) spectra were run. Both mutations in *Tm*GTase caused a perturbation in the structure and dynamics of the protein. The CD spectra of both variants right after purification look similar to the one from WT at 222 nm (Fig. 4a), even though the SDS-PAGE analysis of variant W131G showed a degradation band corresponding to approximately 17% of the total intensity (Fig. 4b). We also observed that this variant was prone to aggregation, especially when maintained in buffer containing 2 mM CaCl_2_, where the CD signal practically disappeared after three days of storage at 4 °C (data not shown). For this reason, we avoided the use of CaCl_2_ in the buffer. In concordance with the fresh-protein CD spectra, the analysis of structures from the MD showed similar secondary structure content for the three proteins (Fig. S3). On the other hand, there is more flexibility in the loop regions for the truncated loop variant. These results are obtained from the calculation of RMSD along the MD simulations (Fig. 4c); the WT protein stabilized close to the starting structure, while both mutants deviate more from their starting structure, and more so for the truncated loop variant. Upon clustering with a 2 Å cutoff over alpha carbons, 99.98% of the conformations of the WT protein can be represented by a single structure (Fig. 4d, left panel). W131G can be represented with one cluster covering 99.44% of the whole population (Fig. 4d, middle panel), while the truncated loop variant experienced more changes during the simulation, as evidenced by a wider distribution of RMSD (Fig. 4c) and the need to include twelve cluster structures to represent 98% of the population (Fig. 4d, right panel). The increase in RMSD and average RMSF per residue for this protein is due to increased motion of the loop in domain B and the residues in a loop across the active site (Fig. 4e–f, rightmost structure). In general, the fluctuation in these loops is heightened in the three variants, but is more prominent for the truncated loop variant, reaching values near 3 Å (Fig. 4e, lower panel).

### The *Tm*GTase Lid is Found in Two Conformations

**Fig. 5:**
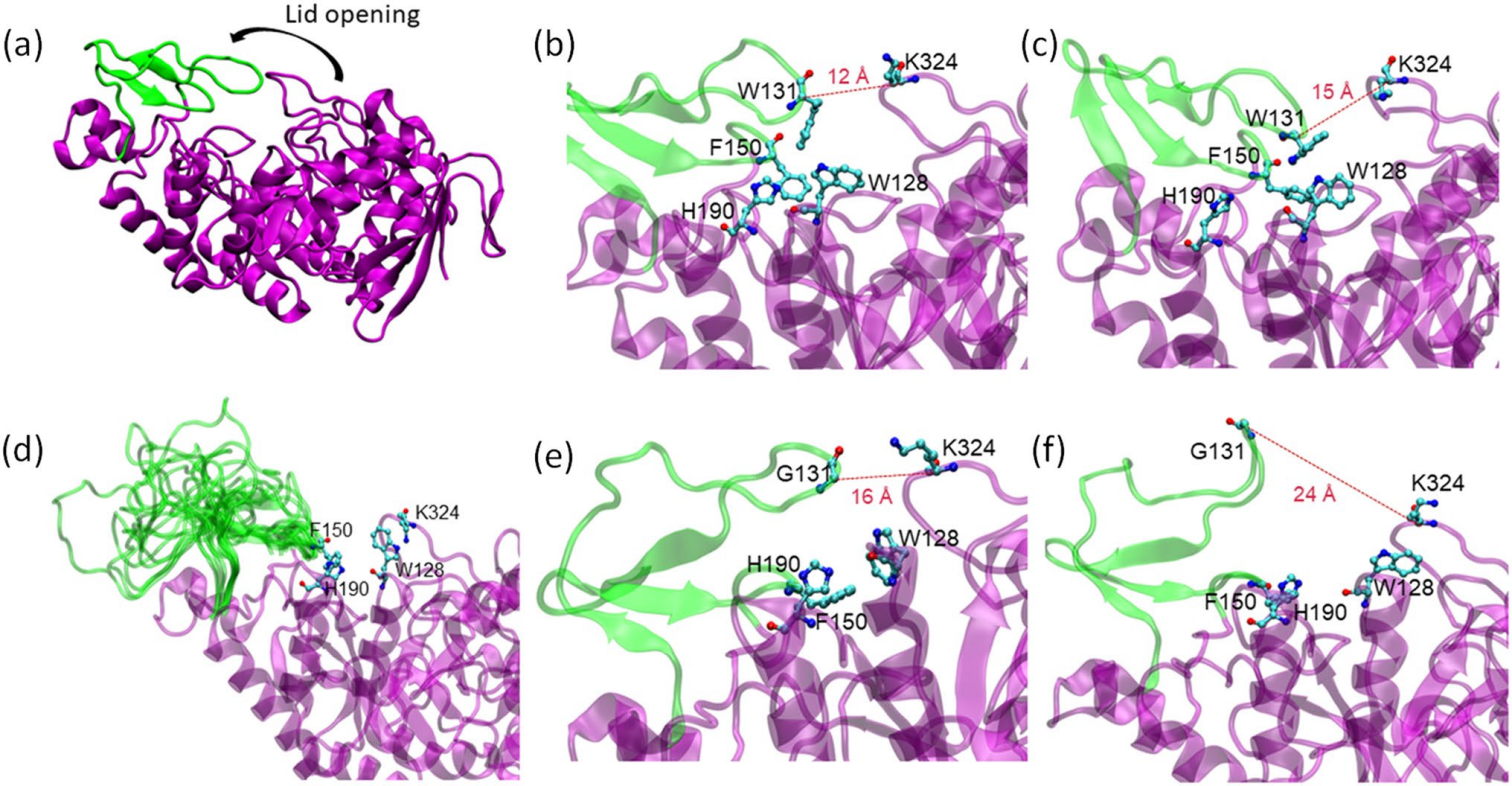
Conformational changes in *Tm*GTase variants. **a** Initial structure from PDB 1LWJ **b** Key interactions in the WT protein result in a closed lid conformation **c** Key interactions in the WT protein result in an open lid conformation **d** truncated loop mutant loses all the WT interactions leading to lid opening and loop disorder **e** Interactions in W131G mutant in closed conformation **f** W131G mutant loses interactions resulting in an open lid conformation

The loop of *Tm*GTase that contains residue W131 is a key structural element to efficiently produce the transglycosylation of starch [29, 65]. Nevertheless, this structural element is not crucial for the hydrolysis reaction. A possible mechanism of catalysis could be associated with the ability of this loop to alternate between open and closed conformations (Fig. 5a). These protein conformations are mediated by the interaction of W131 with K324, W218, H190, and F150 (Fig. 5b–c). The substitution W131G results in a loss of these van der Waals, aromatic, and cation-pi interactions, which favors the open state (Fig. 5e–f). In the extreme case, the truncated loop variant is unable to keep the closed conformation and remains disordered (Fig. 5d).

### Experimental Activity pH Profile

**Fig. 6:**
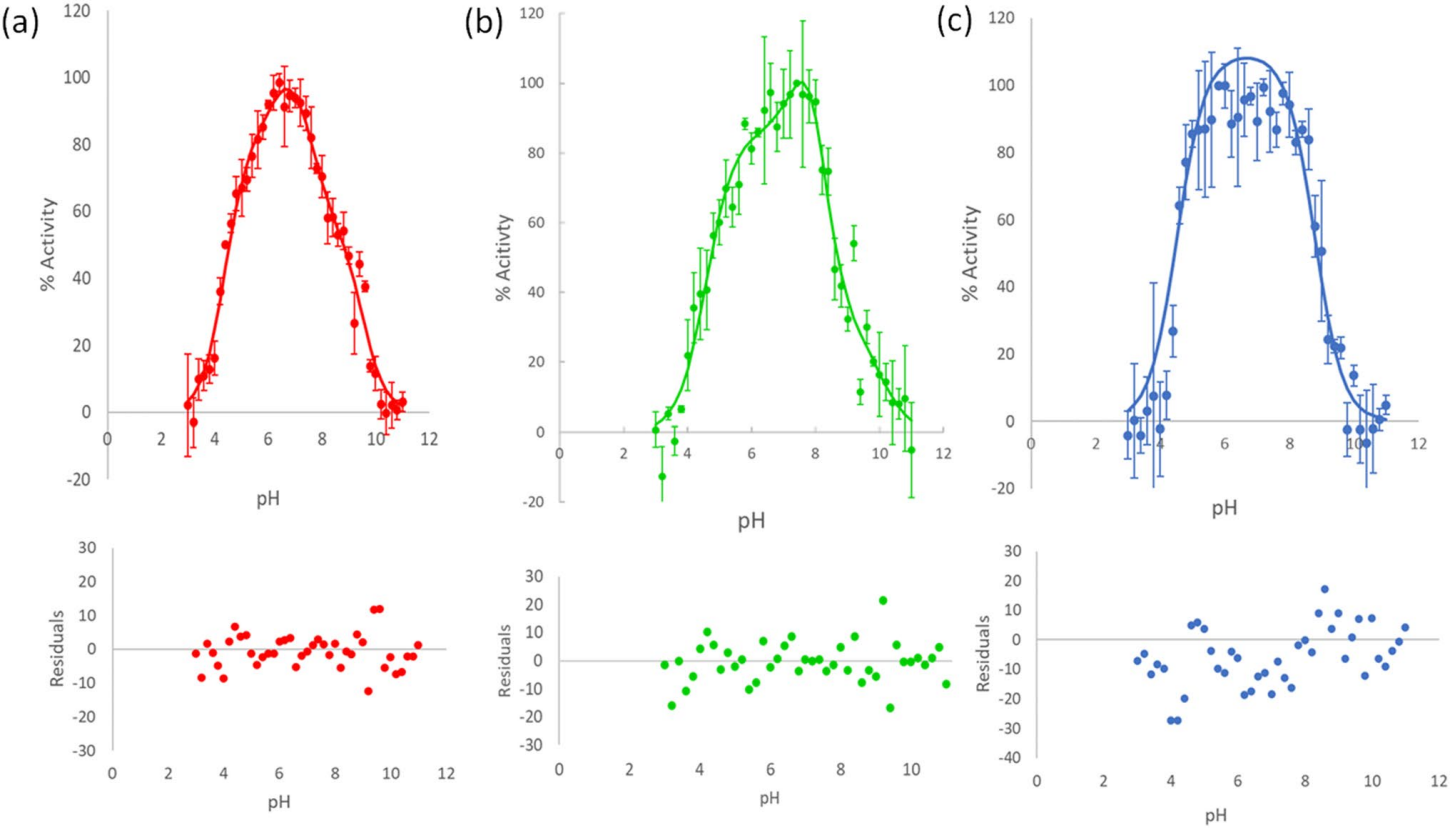
Transglycosylation activity pH profile of *Tm*GTase variants. **a** WT, **b** W131 G, and **c** truncated *Tm*GTase. Data are presented with errors corresponding to 1 SD and the data were fit to Eqs. 3, 3 and 1, respectively shown in the Materials and Methods section. Lower panels show the respective residuals from the experimental data and the model fit

An important property of titratable residues is their p*Ka*, whose value is influenced by the surrounding environment. E216 has been assigned as acid and base in the first and second parts of the reaction, respectively [43]. To determine the effect of the mutations in domain B on E216 and D186 p*Ka*s, the activity pH profile of the WT and variants was measured in the pH range from 3 to 11. As shown in Fig. 6, the WT enzyme showed a complex behavior where the titration of at least two groups on each side of the activity bell can be identified. The fit of the WT and W131G relative activities to Eq. 1, which assumes that the active species contains one active site protonated residue while the other remains deprotonated yields residual errors indicating the presence of multiprotic species (Fig. S4). A more complex model in which the protonation state of other residues influencing the p*Ka* of the active site residues and consequently the protein activity, is proposed to explain the complex pH profile displayed by the WT and W131G proteins, as shown in Fig. 6a, b. In this model, there are three species that show different degrees of activity, with four p*Ka*s listed in Table 3. A curve fitting to a two (Eq. 1) or three (Eq. 2) p*Ka*s shows non-random residuals for both variants (Fig S4). In contrast, the truncated loop pH profile could only be fitted to a model that considers two p*Ka* values.

**Table 3:** p*Ka* of catalytic residues determined from the activity-pH profile

Protein	Fit to equation	p*Ka*_1_	p*Ka*_2_	p*Ka*_3_	p*Ka*_4_	x^c^	y^c^	z^c^	R^2^
WT	1^a^	4.4 ± 0.1	8.9 ± 0.1						0.975
	2^b^	4.4 ± 0.1	6.2 ± 0.7	7.6 ± 0.3	9.5 ± 0.1	81.6 ± 8.9	106.8 ± 10.9	55.8 ± 7.1	0.989
W131G	1^a^	4.7 ± 0.1	8.9 ± 0.1						0.968
	2^b^	4.5 ± 0.09	7.7 ± 0.8	8.1 ± 0.5	10.1 ± 0.4	85.7 ± 4.2	145 ± 62	28 ± 11	0.973
Truncated loop	1^a^	4.6 ± 0.1	8.9 ± 0.1						0.976

p*Ka*_*1*_ corresponds to the nucleophile and the basic limb p*Ka* corresponds to the acid/base residue

^a^Equation 1 consisted of the data fit considering two pKa values

^b^Equation 2 considers four pKa values for three active species

^c^x, y and z are the relative activity for the species

### Dynamics of Acid Residues in the Catalytic Site from MD Simulations

**Fig. 7:**
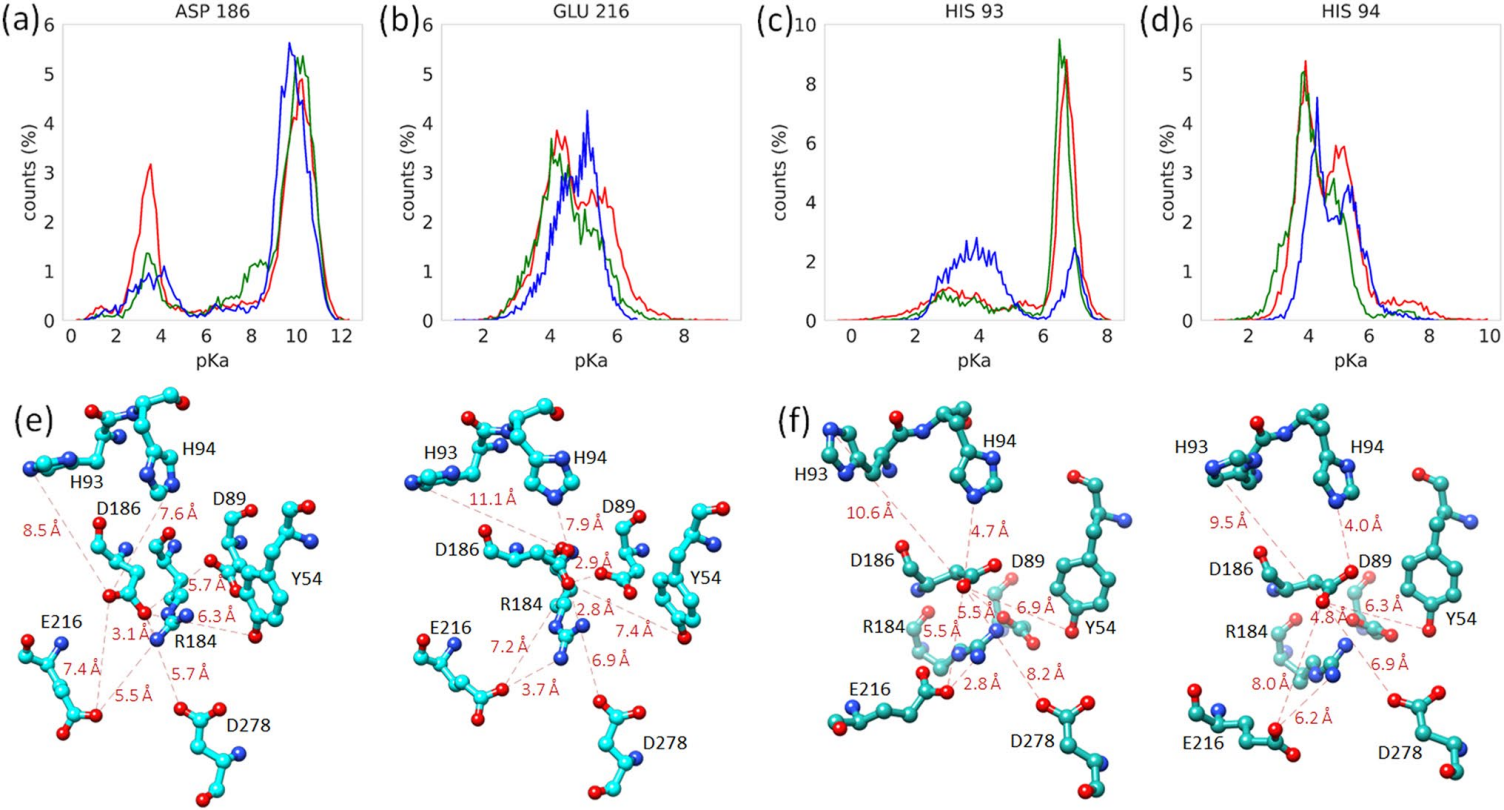
Changes in the computed p*Ka* along the simulation for active site residues. Distribution of p*Ka* values for D186 (**a**), E216 (**b**), H93 (**c**), and H94 (**d**) for WT (red), W131G (green), and truncated loop variant (blue) **e** representative structures of the distribution of D186 with p*Ka* = 3.00 (left panel) and 10.00 (right panel) in WT **f** representative structures of the distribution of truncated loop variant D186 with p*Ka* = 4.00 (left panel) and 6.50 (right panel), respectively. The distances between atoms are represented by broken lines (Color figure online)

The acid-base plasticity of residues located in the catalytic pocket is crucial for the correct functioning of the glycoside hydrolase. The proposed acid-base residue (E216) has a similar, low p*Ka* distribution in the three variants of the *Tm*GTase (Fig. 7b). Surprisingly, D186 alternates between two populations of p*Ka*, one around three and the other near ten, the latter p*Ka* more prominent for all proteins (Fig. 7a). Additionally, the W131G variant shows a small population with D186 p*Ka* around eight. The acid-base residue should be capable of modifying its p*Ka* along the reaction coordinate while the nucleophile p*Ka* should be more stable and remain at least 1.5 units below the acid-base residue p*Ka* [66]. This implies that only the conformations in which D186 has a low p*Ka* could be active. The D186 p*Ka* can be influenced by the electrostatic environment in the active site. One important residue nearby is R184 which organizes the negative charged residues that form the active cleft (D186, E216, D89, D278) (Fig. 7e, f). Interestingly, residue Y54 changes its distance from the catalytic residues depending on the p*Ka* of D186, which suggests a possible role in the functionality of the catalytic pocket. Along the same lines, there are two contiguous histidine residues, H93 and H94, that may contribute to the electrostatic environment in the active site pocket. H93 p*Ka* is centered in 7 and its role may be as a buffer, although it also has a small population with p*Ka* 4 (Fig. 7c). H94 p*Ka*, on the other hand, is distributed between 3 and 6 (Fig. 7d). Representative structures at the extremes of these values indicate that the higher p*Ka* values are compatible with the catalytic acids p*Ka*s for a functional conformation (Fig. 7e, f).

## Discussion

It is recognized that mutations at the active site, especially at the positive substrate binding subsites, influence the reaction specificity as was reported for *Bacillus stearothermophilus* NO2, cyclodextrin glucosyltransferase [11], *B. circulans* CGTase [67] and other CGTases [68]. Some of the amino acids that form these subsites are easily identified in the conserved regions of the TIM barrel catalytic domain; however, some others are not structurally equivalent among homologous enzymes, like those in domain B, which has diverged the most among GH13 enzymes. By comparing the 3D-structures of *Tm*GTase (predominantly transglycosidic) with *Tp*Amylase (one of its closest hydrolase homologous with known structure), an extended loop from domain B with an aromatic residue at the tip was identified only in *Tm*GTase. This structural difference apparently could be associated with the reaction specificities of these GHs. Zebra3D algorithm also identified a larger region (residues 118–154) as relevant for function, which includes this loop. To test our hypothesis, a truncated loop version and the removal of the aromatic residue were investigated. As we predicted, both mutants showed an increased H/T ratio compared to the WT protein. However, the main effect was on the reduction of the transglycosylation reaction. Kinetics analysis showed that the deletion of part of the loop drastically decreased the transglycosidic activity while increasing modestly the hydrolytic one. The importance of loops from domain B lying on the active site has also been observed in Tabium cyclodextrin glycosyltransferase (CGTase), where a loop insertion (characteristic of the hydrolytic G2 amylase) in domain B, placed on subsite − 3/–4, reduced the cyclization/disproportion events (by > 97%) more than the hydrolytic ones (by 56–92%) [37], resulting in a hydrolytic/cyclization ratio increased from 0.23 (WT) to a maximum of 17. Also, van der Maarel´s group [65] reports the role of a flexible loop carrying a Y residue that inserts into the active site. Modification of the loop length has significant effects on the transglycosylation and hydrolysis activities for glycogen branching enzymes (GBEs) from GH family 57, most probably due to displacement of the Y residue.

Notably, the W131 at the tip of the protruding loop of domain B in *Tm*GTase is part of the + 2 subsite, so we decided to investigate its role in the specificity of the reaction. Removal of this aromatic residue drastically decreased transglycosylation activity, although this was not accompanied by a statistically significant (p < 0.1) increase in hydrolysis, like in the truncated loop variant. However, the mutation of this residue had a greater effect on the stability of the protein. It showed some degradation, so the hydrolytic activity of this variant might be underestimated considering its low stability, the high temperature, and the long time needed to measure hydrolysis. We took this into account by measuring the amount of full-length protein at the start of the activity assays and corrected the specific activities accordingly. The importance of aromatic residues in controlling reaction specificity has been demonstrated in many GHs that contain a (β/α)_n_ (n = 7–8) barrel similar to the GH13 family. It has been suggested that their roles could be preventing the entry of water molecules into the active pocket, accommodating the acceptor carbohydrate through π-stacking interactions, and contributing to an active site disposition favorable for transglycosylation. For instance, in the amylomaltase from *T. brockianus* [69] and GTase from *P. furiosus* [70], the presence of aromatic residues in the vicinity of the active site favors the tranglycosylation reaction. In the predominantly transglycosidic GH31 *L. monocytogenes* cycloalternan-forming enzyme (*Lm*CAFE) the modification of W430A increased the hydrolysis from 17 to 97 [29]. In addition, for the GH57 *T. kodakarensis* GBE the Y233A substitution, located in a protruding loop pointing towards the active site, doubled the hydrolytic activity without significant changes in transglycosidic activity [65]. Also, the chemical nature of the residue at the tip of loop affects the substrate and reaction specificities, as was demonstrated with the substitution W324Y in GH31 *Schwanniomyces occidentalis* α-glucosidase [71]. This mutation reduced its hydrolytic capacity and modified its transglycosylation profile. The authors attributed this to the probable different disposition of the substrates around − 1 and + 1 subsites. For *Tm*GTase the W131 residue was shown to contribute to reaction specificity but it also plays a crucial role in protein stability. This residue functions as a clamp that allows the loop to close over the active site. Interestingly and contrary to our expectations, the mutations improved the *Km* for maltose as acceptor in the transglycosylation reaction. In light of this, we propose that the role of the W131 residue, rather than binding the acceptor, could be to orient it so that it remains in a productive conformation. Also, as shown in Fig. 5d–f, removing this residue breaks the interactions that kept the loop closed. This results in a permanent open channel for water molecules to access the active site, as the minimum contact distance between the tip of the long loop and the active site increases from 5 to 10 Å (Fig. S3). The fact that this variant showed a slightly higher hydrolytic activity than the WT enzyme, even though there was a substantial loss of transglycosidic activity, suggests that this enzyme was more suitable for hydrolysis. The increased access of water to the active site accounts for the increased hydrolysis and also for the instability of this variant by exposing the protein interior to the solvent. To our surprise, shortening the loop did not have the catastrophic effect on the stability of the protein in contrast to the single amino acid replacement. This different behavior finds an explanation through the MD, where disorder is observed for the shorter loop in domain B, alternating between open and closed conformations (Fig. 5d). Some research groups have tried to correlate the change in reaction specificity to perturbations in the protein dynamics. However, some studies exhibit no correlation [34], while others show a direct [72] or inverse [36, 73] relationship between reduction in the dynamics of the protein and higher transglycosylation. In an attempt to explain the behavior of the two constructed variants, MD simulation analysis was performed and an increased flexibility in domain B was found for the truncated *Tm*GTase variants. The main effect of the mutations was on maximum velocity (Vmax), with approximately a 10-fold reduction, suggesting that this loop and particularly W131, lock the catalytic configuration. A similar perturbation effect was seen for the *Pyrococcus furiosus* 4-α-glucanotransferase W229H variant (subsite + 1/+2) where a 4.2-fold decrease in the catalytic constant of the transglycosylation reaction was measured, while the *Km* for acceptor increased 1.6 times [70]. Also, in the cycloalternan-forming enzyme from *L. monocytogenes* (*Lm*CAFE), transglycosylation activity was reduced when one of its loops was replaced by the structural equivalent from the cyclo-alternan degrading enzyme from *Trueperella pyogenes* (*Tp*CADE). The authors attributed this behavior to a reverse Koshland**’**s induced fit mechanism, in which residues close to the active site are locked in the active conformation in *Lm*CAFE (transglycosidic enzyme), showing a high affinity for sugars in the + 1 subsite, i.e., no induced fit occurs, and the site is primed for transglycosylation. In contrast, in *Tp*CADE, the residues forming the + 1 subsite are disordered and approach the active site until substrate binding occurs (induced fit). After loop substitution in CAFE, the extra entropic cost to reach an active conformation increases the activation energy for transglycosylation, slowing the reaction rate and allowing the hydrolysis reaction to take place [29].

Overall, the dynamics and disposition of the acid-base residue seem to be a structural element that controls the balance between hydrolysis and transglycosylation. However, other structural factors might govern the efficiency of the hydrolytic step, like the hydrogen bonds network positioning the water in a correct geometry to attack the GEI.

In addition, the dynamics and the surrounding environment also have repercussions on the physicochemical properties of the catalytic residues, such as the p*Ka*, the charge distribution, and the environmental hydrophobicity/hydrophilicity. The modification of these parameters could favor one of the transition states producing hydrolytic or transglycosidic products [28]. The D186 p*Ka* distribution observed during MD shows that this residue can be in alternate conformations with a low (near 3) and high (near 10) p*Ka* for the three variants. From these two distributions only the low p*Ka* ones are suitable for its role as nucleophile. The decreased frequency of the lower p*Ka* population could account for the loss of transglycosidic activity in both mutants. By inspection of the structures during the MD we observed that a low D186 p*Ka*, is sometimes accompanied by high E216 p*Ka*, in agreement with its role as proton donor at the glycosylation step. This fact points to the need for plasticity on the active center to carry out any of these reactions.

The WT and W131G enzyme activities as a function of pH differ from the classical Henderson-Hasselbalch behavior; the residuals show non-random trends when data are fitted to only two p*Ka*s (Fig. S4). This behavior has been observed in other hydrolases [34, 74]. Notably, we obtained p*Ka* values consistent with those observed in the simulation (Table 3; Fig. 7a, b) by assuming various protonation states, each with different specific activities. Although the simulation was performed with the free enzyme, the MD shows the conformational space that is accessible to the protein. The residues that interact directly or through an interaction network to modify the electrostatic environment of the catalytic pocket can be deduced from the analysis of the representative structures at each active site residue p*Ka*. Thus, it is possible to see how Y54 moves away when the p*Ka* of the catalytic residues is appropriate (Fig. 7f). At the same time, R184 approaches the nucleophile (D186) and moves away from the acid-base residue (E216). In contrast, Y54 moves closer to this residue while R184 moves away to interact with E216 when the high p*Ka* conformational interactions for D186 are analyzed (Fig. 7e). The truncated loop variant is the protein with the most significant change in pH profile, in which the intermediate p*Ka* values observed in the WT protein collapsed to the two extreme p*Ka* values, indicating that the network of interactions influencing the p*Ka* behavior of the catalytic residues was perturbed in this variant. In concordance, during MD, E216 shows a narrower p*Ka* distribution. By analyzing the differences in the electrostatic network of this variant around the catalytic site, it can be observed that H94 moves closer to the active site. The presence of H94 might buffer the electrostatic environment around the catalytic residues. In the case of W131G variant, a fit to 4 p*Ka*s is better (Fig. 6b), with the highest p*Ka* shifting to a value of 10.1 compared to 9.5 for WT. Interestingly one intermediate p*Ka* around 8.0 is obtained for this protein that is coincident with a small peak in the D186 p*Ka* distributions of the MD (Fig. 7a), that might be representing a small population of D186 in an unfavorable conformation for a nucleophilic attack. Enzymes are in constant exploration of the conformational space. The activities measured are the average of every enzyme molecule acting simultaneously. Albeit the simplest model to explain the phenomena could be applied, the observation of non-random distribution in the residuals are indicative of other processes going on. A recent QM-MM analysis of the human pancreatic α-amylase suggests a structural equilibrium in which some conformations lead to the formation of competent E-S complexes while others do not [75]. Our results showing different p*Ka* for the active site residues with different activity agree with these findings.

In *Tm*GTase, the structure has been evolutionarily optimized to favor transglycosylation over hydrolysis. As enzymes that work in an aqueous medium, closing the active site is imperative to control the water entrance to the active site. The presence of long loops in domain B may be one of the strategies for this protein to keep water away from the active site. We achieved a change in H/T ratio by increasing the mobility of one of the loops that form part of the lid to close the active site by increasing the water access to the active site and altering the hydrogen-bond network comprising catalytic residues.

## Conclusions

We obtained two *Tm*GTase variants with a modest increased H/T ratio by substitutions/deletions outside the catalytic domain, specifically in domain B. The importance of the aromatic residue at position 131, which is part of the + 2 subsite, was demonstrated by a drastic reduction of the transglycosidic activity, just as the truncation of the loop containing it. Both mutations showed an increase in the H/T ratio. This was mainly due to the loss of transglycosidic activity with only a modest increment in hydrolysis. Interestingly the single mutation of W131 to G had a major effect on the stability of the protein. This effect was overcome by its removal together with other residues in the loop. The results suggest that the loop not only provides the W residue to lock the active site in an active conformation for transglycosylation, but also acts as a lid to prevent water from entering the active site by switching between closed and open conformations. Comparative analysis of the kinetics and dynamics of these variants relative to the WT protein suggests that the combination of flexibility, hydrophobicity/hydrophilicity of the active site, and p*Ka* of the catalytic residues are responsible for the observed changes in reaction specificity. During evolution, the sequence of the WT *Tm*GTase has been optimized to keep the catalytic residues in an optimal configuration to carry out the transfer reactions at the expense of the ancestral hydrolytic activity. Thus, the two mutations in *Tm*GTase seem to interfere with this complex interaction of factors making the transfer to groups other than water less favorable and altering the specificity of the reaction.

## Supplementary Information

Below is the link to the electronic supplementary material.

Supplementary material 1 (DOCX 6761 kb)

## Data Availability

The authors declare that the data supporting the findings of this study are available within the article.
