# Supplementary material for: The Role of a Loop in the Non-catalytic Domain B on the Hydrolysis/Transglycosylation Specificity of the 4-α-Glucanotransferase from Thermotoga maritima
Source: Protein J. 2023 Jul 18;42(5):502–18. doi: 10.1007/s10930-023-10136-2 (PMC10480278; doi:10.1007/s10930-023-10136-2)
Supplement: Supplementary file 1 — Supplementary material 1 (DOCX 6761 kb) [file 10930_2023_10136_MOESM1_ESM.docx]

**Supplementary material**


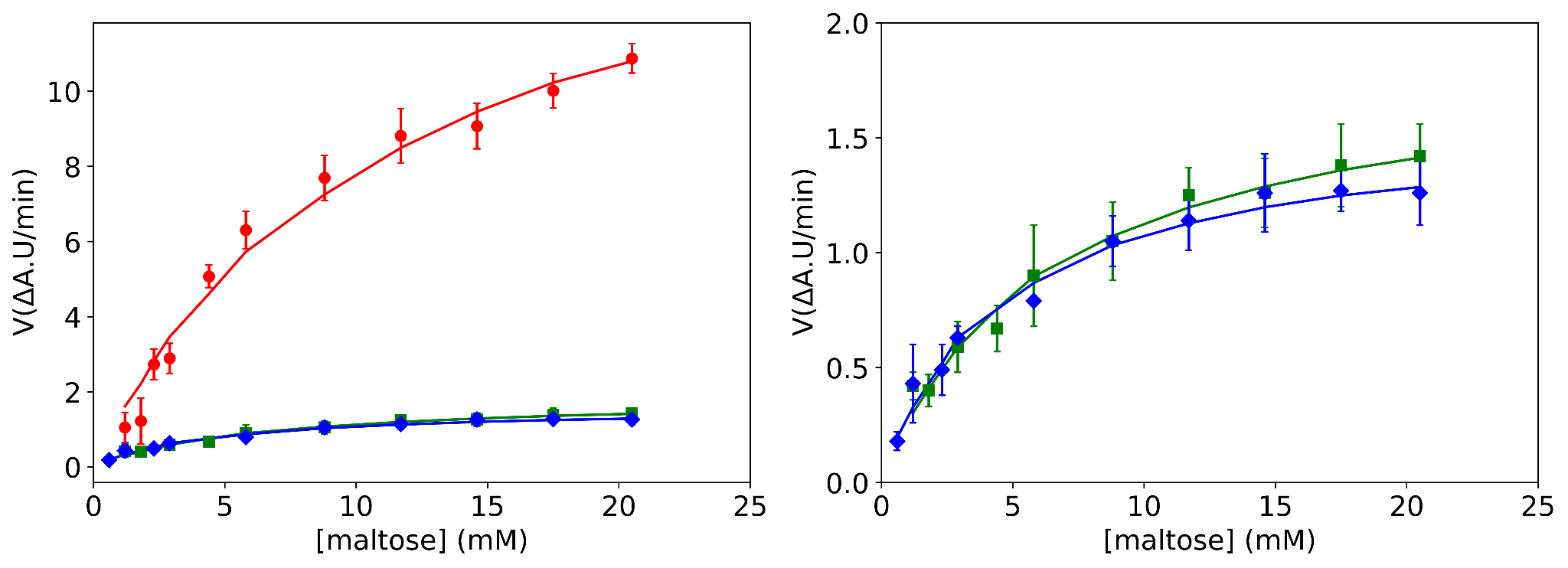


**Fig. S1.** Michaelis-Menten plots for *Tm*GTase transglycosylation reaction keeping constant starch composition and varying maltose concentration. (a) all variants, (b) Enlargement for W131G and shortened loop.


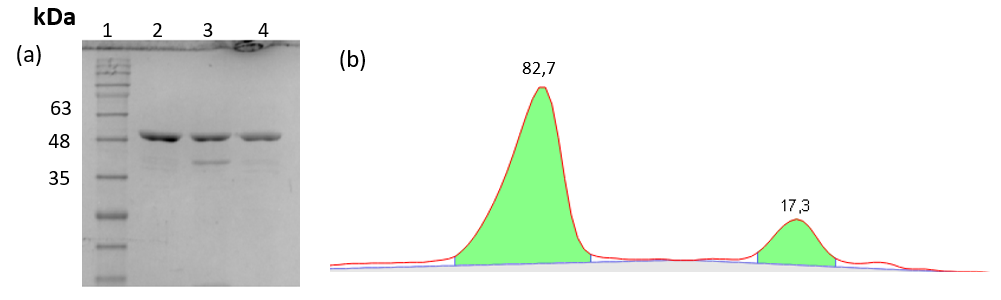


**Fig S2.** (a) SDS-PAGE profile of right purified *Tm*GTase variants. Lane 1, molecular weight marker; lane 2-4, WT, W131G and truncated loop, respectively. (b) Densitometric analysis of lane 3 performed with Image Lab software. Bands are presented in decreasing molecular weight.


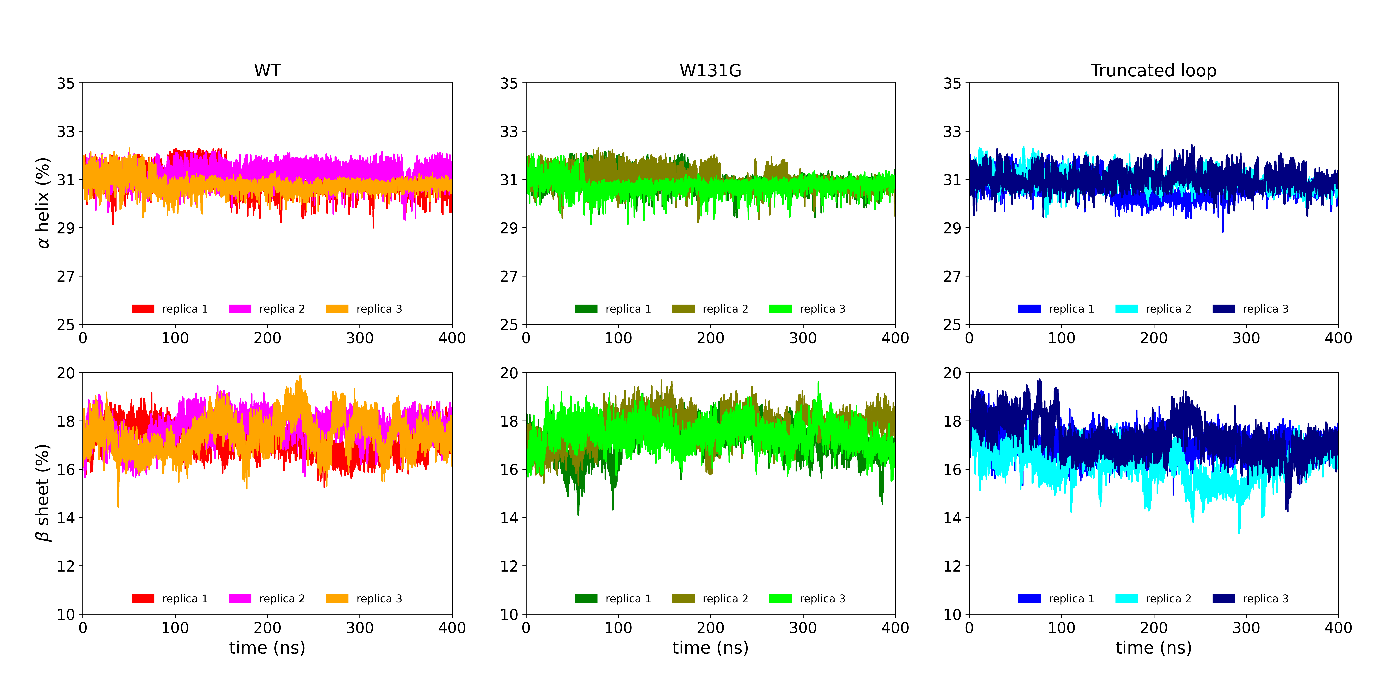


**Fig S3.** The percentage of α-helices (superior panel), and β-sheet (lower panel) for the three variants along three replicas of MD.


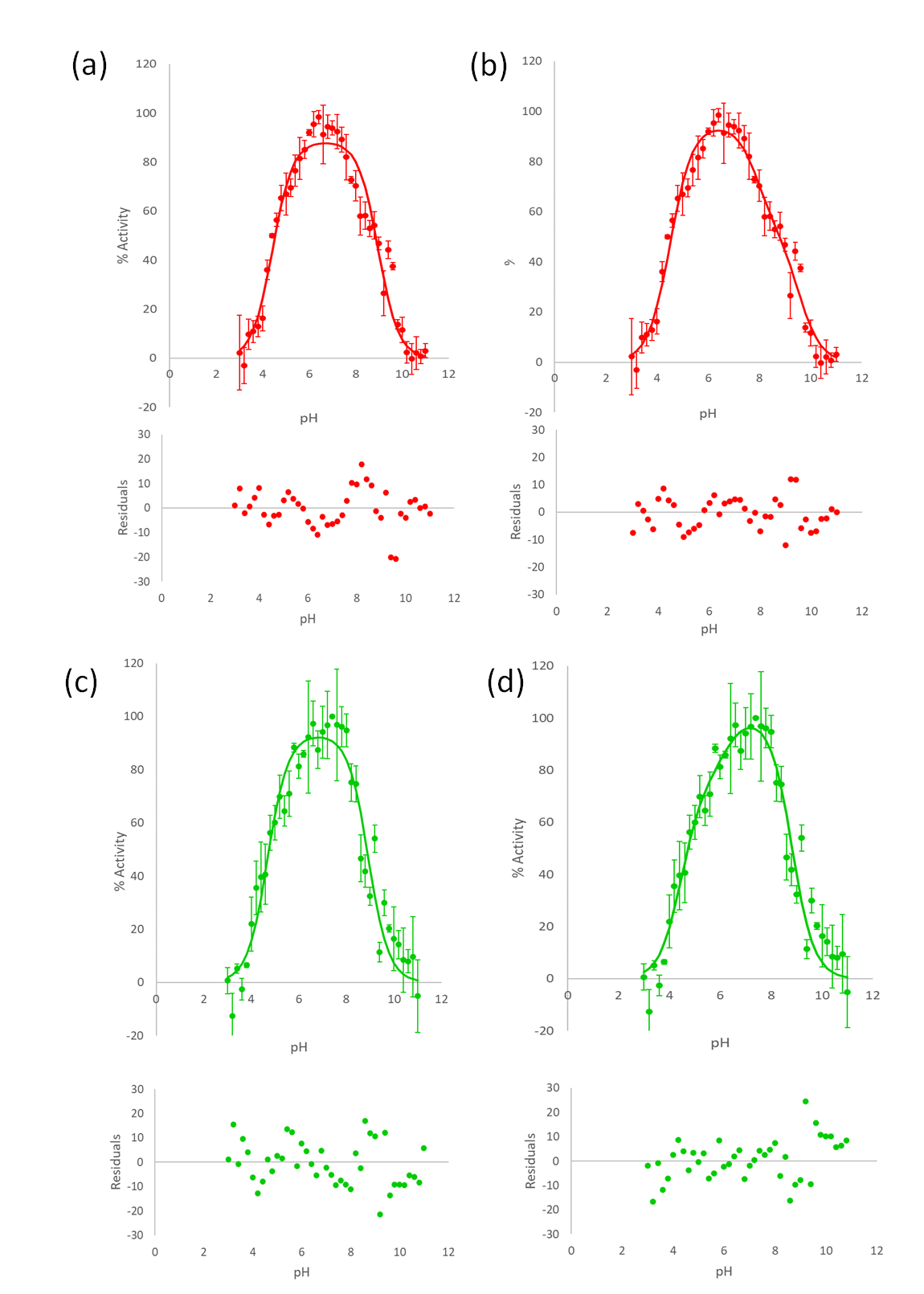


**Fig. S4.** pH dependence of transglycosydic activity for *Tm*GTase mutants fit to two p*Ka*s (a,c) and three p*Ka*s (b,d). WT (red) and W131G (green). The lower panels show the residuals of each model.


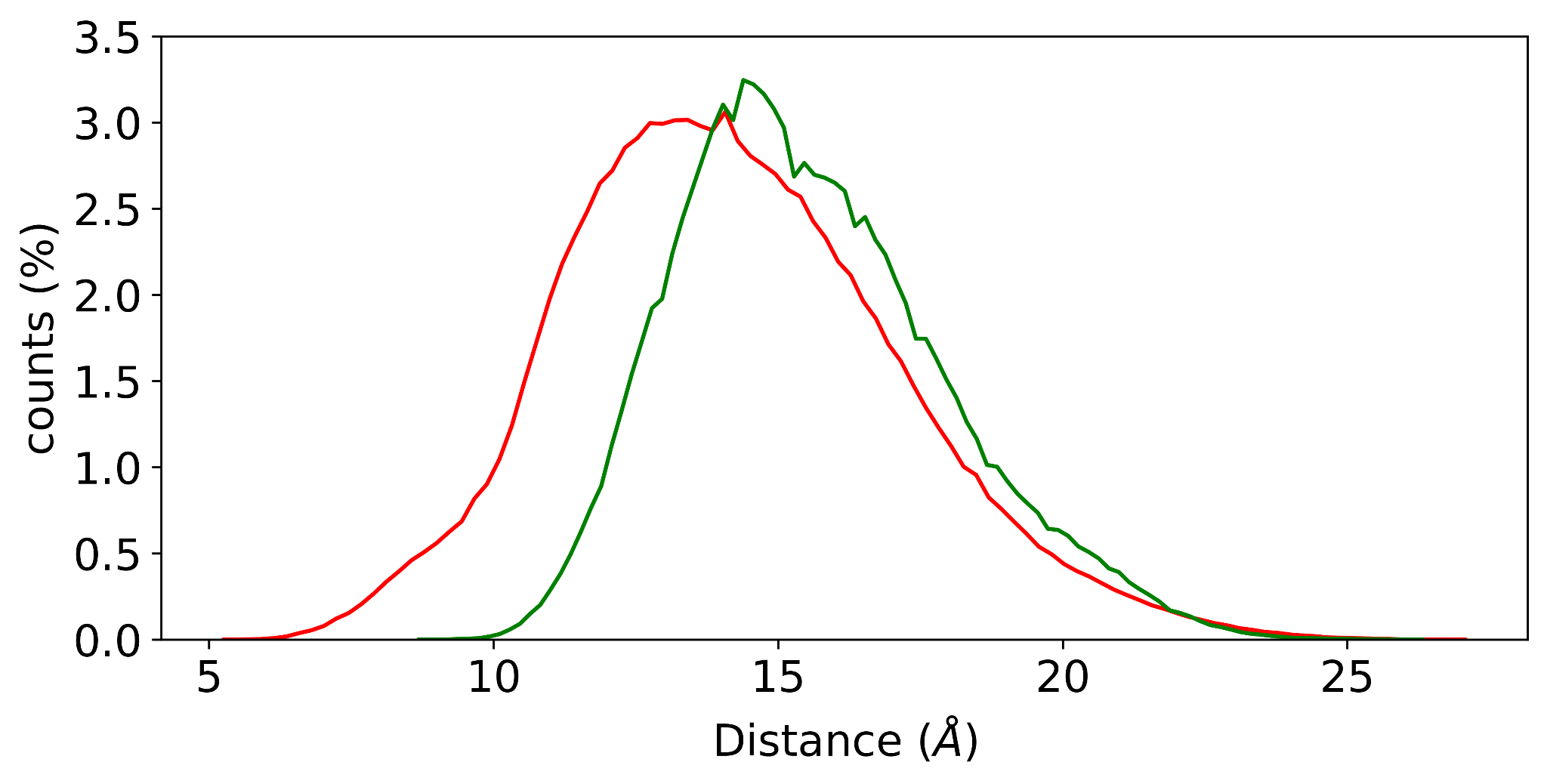


**Fig. S5.** Histogram of the distances between the alpha carbons of residues 131 and 324 for the WT (red) and W131G (green) enzymes.
